# Prävalenz ungewollter Schwangerschaften und Schwangerschaftsausgang bei Frauen mit psychischen Erkrankungen. Ergebnisse aus der ELSA Studie

**DOI:** 10.1055/a-2642-6029

**Published:** 2025-07-22

**Authors:** Silvia Krumm, Yamara-Monika Wessling, Cornelia Helfferich, Maika Böhm, Petra J. Brzank, Ulrike Busch, Daphne Hahn, Christine Knaevelsrud, Tilmann Knittel, Laura Olejniczak, Sarah Schumacher, Susanne Jaeger

**Affiliations:** 1 Klinik für Psychiatrie und Psychotherapie II der Universität Ulm am Bezirkskrankenhaus Günzburg; 2 Klinik und Poliklinik für Psychiatrie und Psychotherapie, Medizinische Fakultät, Universität Leipzig; 3 Sozialwissenschaftliches Forschungsinstitut zu Geschlechterfragen – SOFFI F., Forschungs- und Innovationsverbund an der Evangelischen Hochschule Freiburg (FIVE e.V.), Freiburg; 4 Fachbereich Soziale Arbeit.Medien.Kultur, Hochschule Merseburg, Merseburg; 5 Institut für Sozialmedizin, Rehabilitationswissenschaften und Versorgungsforschung, Hochschule Nordhausen, Nordhausen; 6 Fachbereich Gesundheitswissenschaften, Hochschule Fulda; 7 Fachbereich Erziehungswissenschaft und Psychologie, Freie Universität Berlin; 8 Institute for Mental Health and Behavioral Medicine, HMU Health and Medical University Potsdam; 9 Klinik für Psychiatrie und Psychotherapie I der Universität Ulm, ZfP Südwürttemberg (Weissenau)

**Keywords:** Ungewollte Schwangerschaften, Prävalenz, Schwangerschaftsabbrüche, Soziale Kontextfaktoren, unwanted pregnancies, prevalence, abortion, social context factors

## Abstract

**Ziele:**

Internationale Studien deuten auf ungünstige Wechselwirkungen zwischen ungewollten Schwangerschaften und psychischer Gesundheit. Die Studie untersucht Prävalenz, Ausgang und soziale Faktoren ungewollter Schwangerschaften bei psychisch erkrankten Frauen in Deutschland.

**Methodik:**

In einer Querschnittstudie wurden 4495 Frauen über das Melderegister sowie 478 Frauen über relevante Anlaufstellen rekrutiert und zu Anzahl, Gewolltheit, Schwangerschaftsausgang und sozialen Kontextbedingungen befragt.

**Ergebnisse:**

Frauen mit psychischen Erkrankungen zeigen ein erhöhtes Risiko für ungewollte Schwangerschaften (OR=1,6), Abbrüche (OR=1,6) und ungewollt ausgetragene Schwangerschaften (OR=1,8). Ungewollte Schwangerschaften treten bei Frauen mit psychischen Erkrankungen häufiger in belastenden Lebensumständen auf.

**Schlussfolgerung:**

Die Wechselwirkungen zwischen ungewollten Schwangerschaften, psychischer Gesundheit und sozialen Belastungen verdeutlichen die Notwendigkeit, Aspekte der reproduktiven Gesundheit in der Versorgung von Frauen mit psychischen Erkrankungen zu berücksichtigen.

## Abstract (en)

**Objective:**

International studies suggest an interaction between unwanted pregnancies and mental health. This study examines prevalence, outcomes, and social factors of unwanted pregnancies among women with mental illness in Germany.

**Methods:**

In a cross-sectional study, 4495 women were recruited via the population register and 478 women via relevant contact points and asked about the number, intent, pregnancy outcome and social context.

**Results:**

Women with mental illness showed an increased risk of unwanted pregnancies (OR=1.6), abortions (OR=1.6), and unwanted pregnancies carried to term (OR=1.8), and unwanted pregnancies are more often linked to stressful life circumstances.

**Conclusion:**

Interactions between unwanted pregnancies, mental health, and social stressors underline the need to integrate reproductive health into recovery-oriented care.

## Einleitung

Obwohl die Gesamtzahl nicht intendierter Schwangerschaften aufgrund eines besseren Wissens über Verhütungsmöglichkeiten, des Zugangs zu und der Erreichbarkeit von Beratungsangeboten und der Nutzung von Verhütungsmitteln weltweit zurückgegangen ist 1 , sind sie ein häufiges Ereignis im Leben von Frauen. Schätzungen zufolge sind 40–45% aller Schwangerschaften weltweit unintendiert 2 bei regionalen Unterschieden 3 . Für Deutschland zeigte die Studie *frauen leben 3,* dass 29% der zwischen 1983 und 2020 ausgetragenen oder abgebrochenen Schwangerschaften nicht intendiert waren, die Hälfte davon explizit ungewollt 4 . Frauen mit psychischen Erkrankungen haben im Vergleich zu Frauen ohne psychische Erkrankungen ein erhöhtes Risiko für nicht intendierte (definiert als ungewollte oder zu früh eingetretene) Schwangerschaften (OR 1,34, CI 1,08–1,67) bei einer gewichteten Prävalenz von 65% (CI 0,43–0,82) 5 . Im Rahmen einer Befragung von Müttern mit schizophrenen Erkrankungen (n=192) in Spanien gaben lediglich 25% der Teilnehmerinnen an, dass die Schwangerschaft geplant gewesen sei 6 .

Nicht intendierte Schwangerschaften bergen gesundheitliche Risiken für die Frau wie auch – im Fall des Austragens – für den Fötus bzw. das Kind 7 8 9 10 11 . Frauen mit nicht intendierten Schwangerschaften haben ein erhöhtes Risiko für peri- und postpartale Depressionen 12 13 14 und zeigen ein ungünstigeres Gesundheitsverhalten im Vergleich zu Frauen mit geplanten Schwangerschaften 11 15 . Während lediglich ‚zu früh‘ (früher als geplant eingetretene, in der Regel aber gewollte) Schwangerschaften aus der Sicht betroffener Frauen nicht zwangsweise problematisch sind, werden ungewollte Schwangerschaften überwiegend negativ bewertet 16 . Mütter, die ihre erste Reaktion auf die Feststellung einer nicht beabsichtigten Schwangerschaft retrospektiv als „ambivalent“ oder „negativ“ beschreiben, geben neun Monate nach der Geburt mehr psychosozialen Stress und weniger soziale Unterstützung an im Vergleich zu Müttern mit positiven Reaktionen auf die Schwangerschaft 17 . Für Frauen mit vorbestehenden psychischen Erkrankungen kann der Abbruch einer ungewollten Schwangerschaft mit psychischen Belastungen einhergehen. Es ist wichtig zu betonen, dass psychische Belastungen bei Frauen nach einem Schwangerschaftsabbruch nicht auf das Abbruchsereignis an sich rückführbar sind, sondern maßgeblich durch frühere psychische Belastungen erklärt werden können 18 . Eine prospektive Kohortenstudie aus den Niederlanden wies ein leicht erhöhtes Risiko für rezidivierende psychische Erkrankungen bei Frauen mit psychischen Vorbelastungen nach einem Schwangerschaftsabbruch nach 19 .

Soziale Faktoren spielen bei nicht intendierten bzw. ungewollten Schwangerschaften eine wesentliche Rolle. Ein geringer sozioökonomischer Status, ein niedriger Bildungsstand wie auch ein junges Alter (<20 Jahren) erhöhen das Risiko für nicht intendierte Schwangerschaften 20 . Soziodemografische Faktoren wie Alter, Bildung und Religion können die Entscheidungen hinsichtlich Abbruch oder Austragen nicht intendierter Schwangerschaften bei jüngeren Frauen beeinflussen 2 . Zudem können psychosoziale Stressoren für die Bewältigung nicht intendierter Schwangerschaften und bei der Entscheidung über den Schwangerschaftsausgang bedeutsam sein. Wie eine qualitative Studie aus den USA nahelegt, werden Entscheidungen bezüglich Abbruch oder Austragen einer nicht intendierten Schwangerschaft durch Versuche zur Vermeidung der sozialen Stigmatisierung von Mutterschaft außerhalb normativer Konventionen beeinflusst 21 . Frauen, die sich aufgrund eines Schwangerschaftsabbruchs stigmatisiert fühlen, berichten häufiger von depressiven Symptomen, Angst und Stress im Vorfeld des Eingriffs 22 . Auch im Falle des Austragens einer nicht intendierten Schwangerschaft kann es zu Stigmatisierung kommen. Frauen mit psychischen Erkrankungen erleben in Bezug auf Elternschaft vielfältige Diskriminierungen 23 . Häufig kumulieren bei Frauen mit psychischen Erkrankungen belastende Lebensumstände in Form von geringem sozioökonomischem Status, Einelternfamilien, Partnerschaftskonflikten, Gewalterfahrungen und Stigmatisierungserfahrungen 24 25 26 27 28 29 .

Zusammenfassend stützen die vorgestellten Befunde die Annahme, dass Frauen mit psychischen Erkrankungen einerseits eine höhere Wahrscheinlichkeit für das Eintreten einer ungewollten Schwangerschaft haben und andererseits aufgrund der vorbestehenden psychischen Belastung und weiterer psychosozialer Stressoren einen besonderen Unterstützungsbedarf im Umgang mit einer ungewollten Schwangerschaft aufweisen. Für die Entwicklung angemessener Angebote ist ein grundlegendes Wissen hinsichtlich Prävalenz ungewollter Schwangerschaften bei Frauen mit psychischen Erkrankungen, Schwangerschaftsausgang (Abbruch/Austragen) und sozialer Kontextfaktoren unverzichtbar. Derzeit liegen für Deutschland weder Daten zur Prävalenz von ungeplanten oder ungewollten Schwangerschaften bei Frauen mit psychischen Erkrankungen noch zu den sozialen Kontextfaktoren vor. Das Ziel der vorliegenden Studie ist es, die Forschungslücke zu schließen und folgende Fragestellungen zu untersuchen:

1. Wie hoch ist die Prävalenz ungewollter Schwangerschaften und deren Ausgang bei Frauen mit psychischen Erkrankungen?
2. Wie sind die sozialen Kontextbedingungen beim Eintritt der Schwangerschaft?

## Methodik

### Datenbasis und Zielgruppe

Die Studie ist Teil der vom Bundesministerium für Gesundheit geförderten, multizentrischen Studie ELSA („Erfahrungen und Lebenslagen ungewollt Schwangerer. Angebote der Beratung und Versorgung“ 30 ), die Belastungen und Ressourcen betroffener Frauen sowie Fragen der medizinischen bzw. psychosozialen Versorgung nach Eintritt der ungewollten Schwangerschaft untersucht. Besondere Aufmerksamkeit gilt Frauen mit Gewalt- und Migrationserfahrung, psychischen Belastungen oder Traumatisierungen. Das Teilprojekt ELSA PSY an der Klinik für Psychiatrie und Psychotherapie II der Universität Ulm widmet sich den Erfahrungen und Bedarfen von Frauen mit psychischen Erkrankungen.

Die ELSA Studie folgt einem Mixed-Methods-Design. Zunächst wurde, unterstützt durch das Befragungsinstitut KANTAR, eine bevölkerungsrepräsentative Online-Befragung von Frauen mit mindestens einem Kind unter sechs Jahren im Haushalt durchgeführt. Im zweiten Schritt erfolgten qualitative Vertiefungsstudien. Potenzielle Studienteilnehmerinnen für die quantitative Befragung wurden über Einwohnermeldeämter sowie weitere Anlaufstellen (z. B. Einrichtungen zur Durchführung von Schwangerschaftsabbrüchen, Beratungsstellen) gewonnen. Für die folgende Analyse wurden Datensätze aus zwei Quellen unter Verwendung eines identischen Fragebogens genutzt: 1.) eine für Deutschland repräsentative Online-Befragung über die Einwohnermeldeämter (EMA-Stichprobe) sowie 2.) eine Befragung von Frauen, die über Einrichtungen für Schwangerschaftsabbrüche, Beratungsstellen sowie Social Media oder Women on Web rekrutiert wurden (ABB-Stichprobe). Die Daten wurden zwischen Dezember 2021 und September 2022 erhoben. Es liegt ein positives Votum der Ethik-Kommission der Hochschule Fulda als koordinierendem Zentrum vom 27.04.2021 vor (Az.: 3.1.9.2-kkm).

### Erhebungsinstrumente

Der Online-Fragebogen wurde im Rahmen der multizentrischen ELSA-Studie entwickelt. Mittels einer Schleifenabfrage wurden sämtliche Schwangerschaften in der Lebenszeit mit Jahr, Intention der Schwangerschaft (gewollt oder bereits früher gewollt, erst später gewollt, unentschieden, ungewollt; Kategorien nach 31 ), Verhütungsverhalten sowie Ausgang der Schwangerschaft (Austragung, Abbruch, Fehl- bzw. Totgeburt) erfasst. Die Schwangerschaften wurden anhand der Intention beim Eintritt der Schwangerschaft klassifiziert. Als zusätzliches Kriterium wurde die Anwendung einer als sicher geltenden Verhütungsmethode (nach Pearl Index) verwendet. Als „gewollt“ wurden Schwangerschaften eingeordnet, die explizit als gewollt bezeichnet wurden oder bereits früher hätten eintreten sollen. Als „ungewollt“ wurden Schwangerschaften eingeordnet, die explizit als ungewollt bezeichnet wurden sowie Schwangerschaften, die erst zu einem späteren Zeitpunkt hätten eintreten sollen bzw. Schwangerschaften mit ambivalenter Intention, die trotz Verhütungsmaßnahmen eingetreten waren 31 .

Da der Fokus der Gesamtstudie ELSA auf den sozialen Bedingungen ungewollter Schwangerschaften lag, wurden die Teilnehmerinnen anschließend durch eine Filterführung zu vertiefenden Fragen bezüglich ihrer zuletzt ausgetragenen ungewollten Schwangerschaft (im Sinne der o. g. Definition) geleitet. Im Fall keiner ausgetragenen Schwangerschaft wurde zu Fragen zum zuletzt angegebenen Schwangerschaftsabbruch geleitet, wobei den Frauen freistand, ob sie hierzu Angaben machen wollten. Alternativ oder im Fall ausschließlich gewollter Schwangerschaften wurden die Umstände der aktuellsten gewollten Schwangerschaft erfragt. Diese im Zentrum stehende Schwangerschaft wird im Folgenden als „Fokusschwangerschaft“ bezeichnet. Die Fragen betrafen unter anderem Zeitpunkt des Bemerkens der Schwangerschaft, psychisches und körperliches Wohlbefinden, Stigmatisierungserfahrungen im Kontext der Schwangerschaft, sozialer Kontext und Lebensumstände sowie Erfahrungen in der Versorgung (medizinisch, psychosozial). Angaben zu psychischer Gesundheit umfassten medizinische oder psychotherapeutische Behandlungen aufgrund psychischer Probleme, selbst berichtete Diagnosen (psychiatrische/psychotherapeutische), Thematisierung psychischer Probleme im Rahmen der medizinischen Versorgung bzw. reproduktiver Aspekte im Rahmen der psychiatrischen/psychotherapeutischen Behandlung und eine visuelle Analogskala zu psychischem Befinden.

Es wurden keine klinischen Skalen zur Diagnostik verwendet. Vielmehr wurde das Vorliegen einer psychischen Erkrankung entsprechend dem Selbstbericht der Teilnehmerin operational definiert: a.) eine ärztliche und/oder psychotherapeutische Behandlung aufgrund psychischer Probleme in den letzten zehn Jahren, b.) eine im Rahmen dieser Behandlung gestellte Diagnose einer psychischen Erkrankung nach ICD-10 und c.) die explizite Angabe einer Diagnose anhand eines Menüs mit Mehrfachauswahlmöglichkeit bzw. Freitext. Teilnehmerinnen, auf die sämtliche Bedingungen zutrafen, werden im Folgenden als „Frauen mit psychischen Erkrankungen“ (FmPE) bezeichnet, alle anderen als „Frauen ohne psychische Erkrankungen“ (FoPE). Eine differenzierte Klassifikation nach Anzahl und Schweregrad der Diagnosen erfolgte nicht.

Für den Vergleich der Kontextbedingungen ungewollter Schwangerschaften zwischen den beiden Gruppen (FmPE vs. FoPE) wurden nur Daten von Teilnehmerinnen einbezogen, bei denen die berichtete Schwangerschaft nach 2012 stattgefunden hatte und damit maximal 10 Jahre zurücklag.

### Statistik

Zum Vergleich der Schwangerschaftsbiografien bei FmPE bzw. FoPE wurden die Stichproben aufgrund eines möglichen Selektionsbias getrennt voneinander ausgewertet. Die EMA-Stichprobe umfasste Frauen mit mindestens einem Kind, während die ABB-Stichprobe Frauen mit hoher Wahrscheinlichkeit für ungewollte Schwangerschaften oder Schwangerschaftsabbrüche einschloss. Verglichen wurden die Häufigkeiten gewollter und ungewollter Schwangerschaften sowie ausgetragener und abgebrochener Schwangerschaften über die gesamte Lebenszeit (getrennt nach Datenquelle).

Zum Vergleich der Fokusschwangerschaften wurden FmPE bzw. FoPE hinsichtlich soziodemografischer Merkmale und der Lebenssituation zum Schwangerschaftszeitpunkt analysiert. Beide Datensätze (EMA, ABB) wurden zusammengeführt und getrennt nach gewollten bzw. ungewollten Fokusschwangerschaften ausgewertet.

Unterschiede in Schwangerschaftsbiografien und Fokusschwangerschaften wurden je nach Skalenniveau mit Chi-Quadrat-Tests, Mann-Whitney-U-Tests oder t-Tests analysiert.

## Ergebnisse

### Stichprobe

Nach Bereinigung umfasste die EMA Stichprobe 4495 Teilnehmerinnen, davon 10% (n=454) mit und 90% (n=4041) ohne eine psychiatrische Diagnose in den letzten 10 Jahren. Die ABB-Stichprobe umfasste 478 Teilnehmerinnen, hiervon wurden 27% (n=129) als FmPE klassifiziert, 73% (n=349) als FoPE. FmPE sind damit in der ABB-Stichprobe fast 3-mal so häufig vertreten wie in der EMA-Stichprobe. Bei FmPE dominieren stichprobenübergreifend affektive Störungen (60%), gefolgt von Angststörungen und Reaktionen auf schwere Belastungen (je 31%), Persönlichkeitsstörungen (12%), psychosomatische und Essstörungen (je 9%) sowie Störungen aus dem Schizophreniespektrum und substanzbezogene Störungen (je 2%). Sonstige Störungen wie z. B. ADHS gaben 3% an. Bei den Teilnehmerinnen der ABB-Stichprobe sind sämtliche Diagnosekategorien häufiger vertreten als in der EMA-Stichprobe, insbesondere Belastungs- und Persönlichkeitsstörungen; die Häufigkeitsreihenfolge war in beiden Stichproben annähernd vergleichbar. Da bei den Diagnosen eine Mehrfachauswahl möglich war, weist die Gesamtverteilung jeweils auf eine relativ hohe Komorbidität verschiedener psychischer Störungen hin, insbesondere bei den Teilnehmerinnen der ABB-Stichprobe.

### Schwangerschaftsbiografie

Tab. 1 zeigt die Lebenszeithäufigkeiten von Schwangerschaften und deren Ausgang bei Frauen mit und ohne psychische Erkrankung getrennt nach den beiden Stichproben.

**Table:** **Tab. 1** Lebenszeitprävalenz und Ausgang von Schwangerschaften von Frauen mit und ohne psychische Erkrankungen.

EMA Stichprobe	FoPE (N=4041)			FmPE (N=454)		
	MW (SD)	MD (min–max)	Mind. 1x n (%)	MW (SD)	MD (min–max)	Mind. 1x n (%)
**Schwangerschaften**	2,48 (1,302)	2 (1–12)	-	2,47 (1,440)	2 (1–13)	-
**ausgetragene Schwangerschaften**	1,97 (0,892)	2 (0–10)	4039 (100)	1,84 (0,815)	2 (1–8)	454 (100)
gewollte ausgetragene Schwangerschaften***	1,82 (0,867)	2 (0–10)	3935 (97,4)	1,61 (0,822)	2 (0–4)	425 (93,6)
ungewollte ausgetragene Schwangerschaften ***	0,15 (0,445)	0 (0–7)	489 (12,1)	0,23 (0,519)	0 (0–4)	87 (19,2)
**Tot-/Fehlgeburten**	0,39 (0,755)	0 (0–9)	1137 (28,1)	0,45 (0,864)	0 (0–7)	137 (30,2)
Schwangerschaftsabbrüche*	0,12 (0,385)	0 (0–5)	406 (10,0)	0,17 (0,437)	0 (0–3)	68 (15,0)
				
**ABB Stichprobe**	**FoPE (N=349)**			**FmPE (N=129)**		
	MW (SD)	MD (min–max)	Mind. 1x n (%)	MW (SD)	MD (min–max)	Mind. 1x n (%)
**Schwangerschaften**	2,29 (1,616)	2 (1–10)	-	2,47 (2,069)	2 (1–14)	-
**ausgetragene Schwangerschaften**	0,88 (1,143)	0 (0–5)	162 (46,4)	0,88 (1,146)	0 (0–6)	63 (48,8)
**gewollte ausgetragene Schwangerschaften**	0,70 (0,973)	0 (0–4)	144 (41,3)	0,65 (1,021)	0 (0–6)	51 (39,5)
**ungewollte ausgetragene Schwangerschaften**	0,18 (0,540)	0 (0–4)	44 (12,6)	0,23 (0,552)	0 (0–3)	23 (17,8)
**Tot-/Fehlgeburten**	0,20 (0,536)	0 (0–5)	55 (15,8)	0,33 (0,851)	0 (0–5)	27 (20,9)
**Schwangerschaftsabbrüche**	1,21 (0,595)	1 (1–6)	349 (100)	1,26 (0,745)	1 (1–6)	129 (100)

Anmerkungen: FoPE: Frauen ohne psychische Erkrankung, FmPE: Frauen mit psychischer Erkrankung; EMA Stichprobe: Einwohnermeldeamtsstichprobe; ABB Stichprobe: gezielt rekrutierte Stichprobe; MW: Mittelwert, SD: Standardabweichung, MD: Median, min-max: Minimum und Maximum, mind. 1x N (%): Anzahl und Prozentsatz der Frauen, bei denen das Ereignis mindestens einmal im Leben auftrat. Prüfung auf Unterschiede zwischen den Verteilungen des jeweiligen Ereignisses mittels

Mann-Whitney-U-Test, ** p<0,01, *** p<0,001

In der EMA-Stichprobe berichteten die 4041 FoPE über insgesamt 10 012 Schwangerschaften, die 454 FmPE über 1120 Schwangerschaften (2,48 bzw 2,47 Schwangerschaften pro Frau). 73% der Schwangerschaften von FoPE vs. 65% der FmP waren gewollt und wurden ausgetragen. Der Anteil ungewollt eingetretener, ausgetragener Schwangerschaften betrug bei FoPE 6%, bei FmPE 9%. Der Anteil ungewollter, abgebrochener Schwangerschaften betrug bei FoPE 5%, bei FmPE 7%. Die Häufigkeitsverteilungen bei gewollten und ausgetragenen Schwangerschaften, ungewollten und ausgetragenen Schwangerschaften sowie abgebrochenen Schwangerschaften unterscheiden sich jeweils signifikant bei FoPE und FmPE. Bei 20% der FoPE, aber bei 29% der FmPE trat im Laufe des Lebens mindestens einmalig eine ungewollte Schwangerschaft ein. FmPE haben somit im Vergleich zu FoPE eine signifikant höhere Wahrscheinlichkeit für eine mindestens einmalige ungewollte Schwangerschaft (OR=1,620; 95% CI: 1,305–2,012, p<0,001), ein signifikant höheres Risiko für mindestens einen Schwangerschaftsabbruch (15% vs. 10%; OR=1,577; 95% CI: 1,195–2,082, p<0,01) und für mindestens eine ungewollt eingetretene, ausgetragene Schwangerschaft (19% vs. 12% OR=1,772; 95% CI: 1,338–2,216, p<0,001).

In der ABB-Stichprobe berichteten die 349 FoPE über insgesamt 799 Schwangerschaften, 129 FmPE über 319 Schwangerschaften. Der Anteil gewollter und ausgetragener Schwangerschaften im Laufe des Lebens lag in dieser Stichprobe bei 30% bzw. 26%, der Anteil der ursprünglich ungewollten und ausgetragenen Schwangerschaften lag zwischen 8% und 9%, der Anteil abgebrochener Schwangerschaften betrug 53% bzw. 51%, der Anteil der Fehl- oder Totgeburten 9% bzw. 14%. Die Unterschiede zwischen FoPE und FmPE waren nicht signifikant. Zudem gab es in der ABB-Stichprobe ein vergleichbares Risiko von FmPE und FoPE für die verschiedenen Ereignisse.

### Fokusschwangerschaften

Nach Bereinigung und Zusammenfassung der ursprünglichen Datensätze blieb eine Stichprobe von n=4270 Teilnehmerinnen mit einer maximal 10 Jahre zurückliegenden Fokusschwangerschaft (957 Schwangerschaften waren ungewollt und 3313 gewollt). FmPE waren bei der Befragung und zum Zeitpunkt der Fokusschwangerschaft jünger, seltener berufstätig und hatten eine geringere Schulbildung als FoPE. Sie bezogen häufiger staatliche Unterstützungsleistungen, gaben Sorgen um die Verschlechterung ihrer finanziellen Situation im Falle eines Kindes an und erlebten ihre Wohnsituation zum Zeitpunkt der Schwangerschaft häufiger als mäßig oder schlecht. Sie gaben häufiger an, in keiner festen oder in einer krisenhaften Partnerschaft gelebt zu haben. Zudem bezeichneten sie ihren Lebensstil zum Zeitpunkt des Schwangerschaftseintritts häufiger als ungesund. Die Zahl vorangegangener Schwangerschaften, der Zeitpunkt des Bemerkens der Schwangerschaft sowie das Vorbestehen körperlicher Probleme unterschied sich nicht zwischen FmPE und FoPE Tab. 2 .

FmPE und FoPE, die über eine gewollte Schwangerschaft berichteten, unterschieden sich in weniger Bereichen voneinander. Allerdings berichteten FmPE vergleichsweise häufiger belastende oder fehlende Partnerschaften, ungünstige Wohnsituationen und eingeschränkte körperliche Gesundheit sowie ein ungünstiges Gesundheitsverhalten.

## Diskussion

Unsere Befunde zeigen, dass Frauen mit psychischen Erkrankungen in Deutschland zwar vergleichbar häufig schwanger werden wie Frauen ohne psychische Erkrankungen, gleichzeitig aber eine höhere Wahrscheinlichkeit für ungewollte Schwangerschaften und Schwangerschaftsabbrüche aufweisen. Damit können wir internationale Befunde unterstützen, wonach Frauen mit psychischen Erkrankungen eine signifikant höhere Wahrscheinlichkeit für ungeplante Schwangerschaften haben 5 32 , sowie erstmals differenzierte Aussagen treffen zur erhöhten Prävalenz dezidiert *ungewollter* Schwangerschaften sowie zu Schwangerschaftsabbrüchen bei Frauen mit psychischen Erkrankungen. In Bezug auf die erhöhte Prävalenz ungeplanter bzw. ungewollter Schwangerschaften bei psychisch erkrankten Frauen werden mangelnde Aufklärung und Informationen über Kontrazeptiva 33 34 35 , inkonsistentes Verhütungsverhalten 36 , pharmakodynamische Interaktionen, die die Wirkung von Kontrazeptiva beeinträchtigen 37 , eine über depressive Symptome vermittelte Einschränkung kognitiver und motivationaler Faktoren 38 39 40 , symptombedingtes Risikoverhalten im Rahmen einer Manie 41 oder eine eingeschränkte reproduktive Autonomie im Rahmen von Partnerschaftskonflikten 42 diskutiert. Gegenüber diesen individuellen Faktoren werden die sozialen Kontextbedingungen kaum thematisiert. Dies überrascht angesichts vorliegender Befunde zur Rolle sozialer Risikofaktoren bei ungeplanten bzw. ungewollten Schwangerschaften 43 . Studien der Bundeszentrale für gesundheitliche Aufklärung zu Familienplanung von Frauen ( *„frauen leben“)* und Männern ( *„männer leben“* ) in Deutschland zeigen, dass das Risiko für ungewollte Schwangerschaften bei beruflicher Unsicherheit oder instabilen Partnerschaften ansteigt 16 44 . Berufliche und finanzielle Unsicherheiten zählen auch zu den häufigsten Gründen bei der Entscheidung für einen Schwangerschaftsabbruch (ebd.).

Unsere Befunde zeigen, dass Frauen mit psychischen Erkrankungen sich beim Eintritt einer ungewollten Schwangerschaft häufiger als Frauen ohne psychische Erkrankungen – aber auch häufiger als psychisch erkrankte Frauen mit gewollten Schwangerschaften – in belastenden Lebensumständen befinden in Bezug auf Wohnen, Finanzen, Bildung und partnerschaftliche Unterstützung. Gesundheitliche Belastungen und ein Mangel an finanziellen, sozialen und kulturellen Ressourcen erhöhen aber nicht nur die Wahrscheinlichkeit, dass eine eingetretene Schwangerschaft als „ungewollt“ und damit negativ bewertet wird, sondern können auch deren Bewältigung sowie die Inanspruchnahme von Unterstützungsangeboten erschweren 17 45 46 47 . Umgekehrt können ungewollte Schwangerschaften weitere psychosoziale Belastungen mit sich bringen. Eine niederländische Studie zeigte, dass Frauen mit ungewollter Schwangerschaft im Vergleich zu Frauen mit gewollter Schwangerschaft und Frauen ohne Schwangerschaft signifikant häufiger über psychosoziale Probleme wie Stressreaktionen, Ängste, depressive Verstimmungen und Beziehungsprobleme berichten, insbesondere während und nach der Schwangerschaft 48 . Es ist naheliegend anzunehmen, dass die mit einer ungewollten Schwangerschaft einhergehenden psychosozialen Belastungen eine bereits vorbestehende psychische Erkrankung zusätzlich negativ beeinflussen.

**Table:** **Tab. 2** Lebensumstände zum Zeitpunkt der ungewollten/gewollten Fokusschwangerschaft von Frauen mit und ohne psychische Erkrankungen.

	Ungewollt			Gewollt		
	FoPE (N=775)	FmPE (N=182)		FoPE (N=3017)	FmPE (N=296)	
Alter bei Befragung MW (SD)	33,3 (6,57)	31,3 (6,43)	***	36,0 (4,58)	36,5 (4,61)	n.s.
Alter bei Befragung MD (min-max)	34 (17–53)	31 (18–46)	***	36 (19–56)	36 (25–49)	n.s.
Alter bei Fokusschwangerschaft MW (SD)	30,9 (6,15)	29,1 (6,14)	***	33,4 (4,15)	33,7 (4,29)	n.s.
Alter bei Fokusschwangerschaft MD (min–max)	31 (17–47)	29 (15–43)	***	33 (18–50)	34 (20–45)	n.s.
Fokusschwangerschaft ausgetragen	52,1%	40,7%	**	(−)	(−)	
abgebrochen	47,9%	59,3%				
Bemerken der Schwangerschaft bis zur 6. Woche	79,1%	84,0%	n.s.	(−)	(−)	
Bildungsindikator (höchster Schul-/Berufsabschluss) „niedrig“	11,7%	20,9%	**	3,9%	5,4%	n.s.
Haupttätigkeit erwerbstätig (vs. Ausbildung, arbeitslos, Hausfrau)	66,4%	51,1%	***	88,1%	85,2%	n.s.
Haushalt bezieht Unterstützungsleistungen	21,0%	29,1%	*	5,9%	7,8%	n.s.
Sorge um finanzielle Situation	32,6%	45,0%	**	5,1%	7,8%	n.s.
Feste Partnerschaft	88,7%	82,9%	*	99,3%	99,0%	n.s.
Partnerschaft fehlend oder krisenhaft	30,0%	41,1%	**	5,1%	8,8%	**
Ungünstige Wohnsituation	33,8%	48,9%	***	13,5%	23,0%	***
Körperliche Probleme>6 Monate	9,0%	13,2%	n.s.	7,2%	15,2%	***
Eher ungesunder Lebensstil	25,7%	39,2%	***	10,2%	15,2%	**

Anmerkungen: FoPE: Frauen ohne psychische Erkrankung, FmPE: Frauen mit psychischer Erkrankung; MW: Mittelwert, SD: Standardabweichung, MD: Median, min-max: Minimum und Maximum. Prüfung auf Häufigkeitsunterschiede mittels Chi-Quadrat-Tests, auf Unterschiede in den Verteilungen mittels T-Tests und Mann-Whitney-U-Tests, * p<0,05, ** p<0,01, *** p<0,001

Reproduktive Gesundheit ist eng mit soziokulturellen Faktoren verknüpft. Gesellschaftliche Vorstellungen zu Geschlecht, Elternschaft, Kindeswohl, aber auch zu psychischen Erkrankungen prägen den individuellen Umgang mit reproduktiven Fragen. Frauen mit psychischen Erkrankungen und ungewollten Schwangerschaften sind mehrfachen Stigmatisierungsrisiken ausgesetzt: Zu dem generellen Stigma psychischer Erkrankungen kommt das Stigma ungeplanter Schwangerschaften 49 sowie (im Fall des Austragens) das mit Mutterschaft im Kontext psychischer Erkrankungen einhergehende Stigma 23 50 51 52 53 oder das Stigma des Schwangerschaftsabbruchs 54 hinzu. Bekanntlich kann die Wahrnehmung von Stigma zu Selbststigmatisierung, geringem Selbstwertgefühl, sozialem Rückzug und erschwertem Zugang zu Hilfsangeboten führen, und damit die psychische und physische Gesundheit zusätzlich belasten und soziale Teilhabe erschweren 55 .

### Limitationen

Die Verteilung der Diagnoseangaben weist darauf hin, dass von den in dieser Studie erreichten Frauen, die wir als „Frauen mit psychischer Erkrankung“ anhand bestimmter Kriterien operational definiert haben, nicht generell auf sämtliche Frauen psychischer Erkrankung geschlossen werden sollte. Die Beantwortung der umfangreichen Befragung im Rahmen der ELSA-Studie setzte ein gewisses Maß an Belastbarkeit voraus, sodass Frauen mit schweren Erkrankungen möglicherweise nicht daran teilgenommen haben. Aufgrund der Rekrutierungswege ist mit einem weiteren Selektionsbias zu rechnen: Beispielsweise sind in der EMA-Stichprobe von Frauen mit Kindern im eigenen Haushalt Frauen mit psychischen Erkrankungen vermutlich unterrepräsentiert, etwa aufgrund des Verzichts auf Mutterschaft oder weil sie nicht mit den Kindern zusammenleben. Bei der Interpretation der Befunde ist weiterhin zu berücksichtigen, dass das Vorliegen einer psychischen Erkrankung operational definiert wurde. Die Zuordnung der Teilnehmerinnen zu den Gruppen FmPE oder FoPE erfolgte nicht auf Basis externer Einschätzung oder klinischer Fragebögen, sondern durch Selbstauskünfte der Teilnehmerinnen anhand weniger Items. Wünschenswert wäre gewesen, hinsichtlich der Diagnosen, ihrer Vollständigkeit und des Schweregrads der Erkrankung genauer differenzieren zu können. Dies bleibt ein Forschungsdesiderat für künftige Untersuchungen. Zudem ist von einem Dunkelfeld tatsächlich psychisch erkrankter Frauen auch in der Gruppe der FoPE auszugehen, da nicht alle Menschen mit einer psychischen Störung Hilfe in Anspruch nehmen und somit trotz bestehender klinisch relevanter Symptomatik häufig keine Diagnose vorliegt. Die tatsächliche Prävalenz ungewollter Schwangerschaften bei Frauen mit psychischen Erkrankungen bleibt daher nur eingeschränkt schätzbar.

Weitere Limitationen ergeben sich bezüglich des Konstrukts „Gewünschtheit“ einer Schwangerschaft. Zum einen lässt sich die Komplexität von Gewünschtheit mittels einer dichotomen Zuordnung „gewollt“ vs. „ungewollt“ nicht hinreichend abbilden (vgl. 15 ). Zum anderen sind die Ergebnisse aufgrund der bis zu 10 Jahren zurückreichenden retrospektiven Einordnung der Gewünschtheit limitiert. Die Ergebnisse können durch Recall Bias und ex-post Rationalisierungen, z. B. auch aufgrund erkrankungsspezifischer Kognitionen, beeinflusst sein. Vorliegende Studien stützen jedoch die Annahme, dass es bei ausgetragenen Schwangerschaften eher zu einer retrospektiven Überschätzung von Gewolltheit kommt 56 . Demnach wäre davon auszugehen, dass die tatsächliche Prävalenz ungewollter Schwangerschaft bei Frauen mit psychischen Erkrankungen höher liegt als es die Befunde unserer Studie nahelegen. Inwieweit umgekehrt eine potenzielle Selbststigmatisierung psychisch erkrankter Frauen die nachträgliche Überschätzung der Ungewolltheit befördert, wäre Gegenstand weiterer Untersuchungen. Schließlich kann nicht ausgeschlossen werden, dass retrospektive Bewertungen einer Schwangerschaft auch durch gesellschaftliche Entwicklungen (z. B. Prävalenz von Schwangerschaftsabbrüchen und deren Versorgung) sowie den Diskursen zu reproduktiven Rechten beeinflusst sind 57 . Dies wäre ebenfalls Gegenstand weiterer Studien.

## Fazit

Reproduktive Gesundheit umfasst laut der Weltgesundheitsorganisation (WHO) die Fähigkeit zu einem verantwortlichen, befriedigenden Sexualleben und zur Fortpflanzung sowie die Freiheit, über Anzahl, Abstand und Zeitpunkt von Geburten selbst zu entscheiden, und auch die Möglichkeit, gewollte Schwangerschaften zu realisieren und ungewollte zu vermeiden 58 . Diese Möglichkeiten werden wesentlich durch soziale Kontextfaktoren bestimmt. In Abgrenzung zu der vorwiegend individualisierenden Diskussion um ungewollte bzw. ungeplante Schwangerschaften im Kontext psychischer Erkrankungen verweist unsere Studie auf die enge Verknüpfung von psychischer Gesundheit, ungewollter Schwangerschaft und prekären Lebensumständen. Angesichts der komplexen Wechselwirkungen sollten Aspekte der reproduktiven Gesundheit stärker als bisher im Rahmen einer recoveryorientierten Versorgung berücksichtigt und Unterstützungsangebote zu Elternschaftsentscheidungen angeboten werden 59 60 61 62 .

## Konsequenzen für Klinik und Praxis

- Frauen mit psychischen Erkrankungen werden genauso häufig schwanger wie Frauen ohne psychische Erkrankungen, berichten jedoch häufiger von ungewollten Schwangerschaften und Abbrüchen.
- Frauen mit psychischen Erkrankungen leben bei Eintritt einer ungewollten Schwangerschaft häufiger unter sozial belastenden Bedingungen.
- Aufgrund der wechselseitigen Zusammenhänge zwischen ungewollter Schwangerschaft, psychischer Gesundheit und sozialer Lage sollte reproduktive Gesundheit als Teil einer recoveryorientierten Behandlung berücksichtigt werden.

## Fördermittel

Bundesministerium für Gesundheit — http://dx.doi.org/10.13039/501100003107; ZMII2–2520FSB11F
